# Circulating extracellular vesicles predict outcome in patient undergoing transjugular intrahepatic portosystemic shunt (TIPS) placement

**DOI:** 10.1038/s41598-025-20562-0

**Published:** 2025-10-07

**Authors:** Sven H. Loosen, Frederik J. Hansen, Denis Schatilow, Theresa Wirtz, Philipp A. Reuken, Johannes Bode, Anselm Kunstein, Mihael Vucur, Johanna Reißing, Georg Flügen, Mirco Castoldi, Andreas Stallmach, Tom Luedde, Tony Bruns, Christoph Roderburg

**Affiliations:** 1 Department of Gastroenterology, Hepatology and Infectious Diseases, University Hospital Düsseldorf, Medical Faculty of Heinrich Heine University Düsseldorf, 40225 Düsseldorf, Germany; 2 Center for Integrated Oncology Aachen-Bonn-Cologne-Düsseldorf (CIOABCD), Aachen, Germany; 3 Department of Medicine III, University Hospital RWTH Aachen, Pauwelsstrasse 30, 52074 Aachen, Germany; 4 Department of Surgery, University Hospital Düsseldorf, Medical Faculty of Heinrich Heine University Düsseldorf, 40225 Düsseldorf, Germany; 5 Department of Internal Medicine IV, University Hospital Jena, Am Klinikum 1, 07747 Jena, Germany

**Keywords:** EV, TIPS, Portal hypertension, Liver cirrhosis, Prognostic markers, Portal hypertension, Translational research

## Abstract

Portal hypertension is a primary cause of complications leading to significant morbidity and mortality in patients with cirrhosis. Transjugular intrahepatic portosystemic shunt (TIPS) insertion has improved survival in well-selected patients with refractory ascites and high-risk variceal bleeding. We investigated the prognostic role of circulating extracellular vesicles (EVs), which are known for their role in immunomodulation and intercellular communication, in patients undergoing TIPS. 141 patients undergoing TIPS placement were included in this retrospective analysis. Median EVs size (X50) and total serum concentration were determined by nanoparticle tracking analysis (NTA) prior to TIPS placement, and transplant-free 1-year survival was assessed using time-to event analysis and Cox regression. EVs size but not their concentration moderately correlated with MELD and Child–Pugh scores based on its correlation with bilirubin and international normalized ratio. In addition, a significant correlation of EVs concentration with platelet count and the immune activation marker soluble urokinase plasminogen activator receptor was observed. In univariate analysis, larger EVs size (> 243 nm) was associated with 1-year transplant-free survival after TIPS placement (*p *= 0.012; HR: 2.539), which remained significant after adjusting for MELD scores in multivariable Cox-regression analysis (*p *= 0.033; HR: 2.204). Larger EVs size indicates advanced stages of chronic liver disease and served as an independent predictor of transplantation-free survival after TIPS placement. Larger prospective studies are needed to confirm these findings and to identify patients at particularly high-risk following TIPS placement.

## Introduction

Portal hypertension (PH) is the primary factor contributing to complications that cause morbidity and mortality in cirrhotic patients^1^. It leads to the development of portal-systemic collaterals, as well as gastric and oesophageal varices, and is associated with a poorer prognosis. Several complications may occur, including bleeding from varices, ascites, and hepatic encephalopathy^2^. In addition, complications related to circulatory dysfunction may occur, such as hepatorenal syndrome, portopulmonary syndrome, and hepatopulmonary syndrome^3^. The introduction of the transjugular intrahepatic portosystemic shunt (TIPS) into clinical practice has been one of the most significant advances in the management of complications of PH^4^. In cases of recurrent or refractory ascites, patients treated with TIPS have better survival than those who receive large volume paracentesis and albumin infusion^5^. In patients with high risk variceal bleeding, preemptive TIPS placement is superior to conservative management and improves survival^6^. However, PH is not the only cause of complications; increased systemic inflammation is also associated with decompensation, particularly in cases of acute-on-chronic liver failure (ACLF)^2,7^. Even when PH is effectively managed after TIPS, liver-derived inflammation appears to be the primary factor leading to organ failure and decompensation in cirrhotic patients^8^. Various methods have been proposed to assess hepatic and systemic inflammation, but individual markers do not appear to reliably predict outcomes in acute decompensation^9^.

Extracellular vesicles (EVs) are small lipid membrane structures released into the surrounding extracellular environment by almost all cell types^10^. They have attracted worldwide interest due to their ability to carry specific cargoes, including proteins and nucleic acids^11^. EVs are important mediators of intercellular communication and can play a critical role in modulatory immune responses^12,13^. However, their potential prognostic value in patients undergoing TIPS placements has not been systematically analyzed.

The aim of this study was to evaluate the potential predictive and prognostic significance of circulating extracellular vesicles as a novel biomarker in patients undergoing TIPS placement for portal hypertension.

### Patients and methods

#### Patient characteristics and study design

This is a retrospective analysis of 141 patients with cirrhosis and severe portal hypertension scheduled for TIPS insertion using ePTFE-covered stents were enrolled at the Jena University Hospital (Germany) between October 2013 and September 2022 and at the University Hospital RWTH Aachen (Germany) between August 2019 and May 2023. This study involves the validation cohort of the previously publishes study^14^; Because serum from 9 patients was unavailable, we included 141 instead of 150 patients. The inclusion criteria were as follows: (1) age between 18 and 85 years, and (2) decompensated cirrhosis (ascites or variceal bleeding) with an indication for TIPS. Exclusion criteria included clinically assessed contraindications for TIPS placement, including severe heart failure, severe pulmonary hypertension, active systemic infection, spontaneous bacterial peritonitis, overt hepatic encephalopathy, or other medical conditions that would make the procedure technically unfeasible. Patients were followed until death or liver transplantation. The present study was performed in accordance with the Declaration of Helsinki. All protocols were approved by the ethics committee of the Jena University Hospital (No. 3683–02/3, 2019–1510, 2018–1080-BO) and the University Hospital RWTH Aachen (No. EK023-19). Written informed consent was obtained from each of the participating patients prior to intervention.

#### TIPS procedure

The TIPS procedure (8–10 mm VIATORR, W.L. Gore, Newark, Delaware, US) was carried out following local standard operating procedures as clinically indicated and previously described^14,15^. Serum samples from the cubital vein were allowed to clot at room temperature for 30 min, then centrifuged at 1000×*g* for 10 min, and stored at − 80°C until further analysis. The median interval between blood collection and TIPS insertion was 0 days (IQR 0–1).

### Measurement of EVs size and concentrations

The size distribution and concentration of EVs were measured using the ZetaView multi-parameter Particle Tracking Analyzer (ParticleMetrix, Germany), as previously described^16,17^. This technique, based on Brownian motion, allows for the analysis of nanometer-sized particles^18^. To ensure accuracy, the ZetaView was auto-aligned before each measurement using a standard calibration nanoparticle solution (110 nm diameter) provided by ParticleMetrix. The camera focus was adjusted to make the particles appear as sharp dots prior to analysis. The sample with the highest anticipated vesicle concentration was used to set the camera sensitivity, which remained constant throughout subsequent measurements. Samples were diluted in particle-free PBS to achieve a particle concentration between 1 and 9 × 10^7 particles/mL (approximately 200 particles per visual field). For each sample, three 30-s videos were recorded using the script control function, with a 5-s pause between recordings and a sample advance between each video.

### Statistical analysis

Statistical analyses were performed as previously described^14,19^. The Shapiro–Wilk test was used to assess normality. Non-parametric data were analyzed using the Kruskal–Wallis ANOVA test. Box plots represent the median, quartiles, and ranges. For the correlation of two variables Spearman´s rank correlation coefficient was applied. The prognostic significance of EVs characteristics and the MELD score was further evaluated through univariate and multivariate Cox regression analyses, including parameters with a *p* value < 0.20 from univariate analysis in the multivariate model. Hazard ratios (HR) with 95% confidence intervals are reported. Kaplan–Meier curves were used to illustrate the effect of EVs parameters on transplantation (TX)-free survival and statistical differences were assessed using the log-rank test. The optimal cut-off value for identifying patients with reduced TX-free survival was determined by fitting Cox proportional hazards models to dichotomized survival status and survival time, defining the cut-off as the point with the most significant log-rank test split^20^. All statistical analyses were performed using SPSS 23 (SPSS, Chicago, IL, USA) and RStudio 1.2.5033 (RStudio Inc., Boston, MA, USA). Differences were considered statistically significant at *p* < 0.05 (* *p* < 0.05; ** *p* < 0.01; *** *p* < 0.001).

## Results

### Characteristics of the study cohort

A total of 141 patients were included in the study. The median age was 60 years, with the youngest patient being 24 years and the oldest 84 years. 78% of the patients were male. The most common indication for TIPS placement (85%) was refractory or recurrent ascites. The most common cause of chronic liver disease was alcohol-associated liver disease (ALD) with 111 patients, followed by metabolic-dysfunction associated steatotic liver disease (MASLD) with 11 patients. 4 patients suffered from cholestatic liver disease and 2 had viral liver disease. Two thirds of patients were in Child–Pugh class B, while 27.7% of the patients were Child–Pugh C and 6.4% of patients were Child–Pugh A. Table 1 summarises the baseline characteristics of the study population.

**Table 1:** Clinical characteristics of the study cohort.

	Patients undergoing TIPS
Number of patients (n)	141
Age (years, median and IQR)	60 (24–84)
Sex, n (%)	
Female	31 (22.0)
Male	110 (78.0)
TIPS indication, n (%)	
Ascites	120 (85)
Bleeding	21 (15)
Etiology, n (%)	
ALD	111 (78.7)
MASLD	11 (7.8)
Cholestatic	4 (2.8)
Viral	2 (1.4)
Other	13 (9.2)
Child–Pugh, n (%)	
A	9 (6.4)
B	93 (66.0)
C	39 (27.7)
Sodium (mmol/L, median and IQR)	135 (122–144)
Creatinine (mg/dL, median and IQR)	0.93 (0.39–7.17)
Bilirubin (mg/dL, median and IQR)	2.0 (0.4–6.98)
Albumin (g/dL, median and IQR)	2.9 (1.8–4.0)
INR (mmol/L, median and IQR)	1.3 (1.0–3.5)
Platelets (× 1000/µL, median and IQR)	145 (17–455)
ALT (U/L, median and IQR)	38 (9–437)
WBC (× 1000/µL, median and IQR)	6.4 (1.0–25.3)
CRP (mg/L, median and IQR)	12.8 (0.3–146.9)
Soluble urokinase plasminogen activator receptor (suPAR) (ng/mL, median and IQR)	8.8 (6.5–11.2)

### The median particle size is associated with advanced Child–Pugh class

**Fig. 1:**
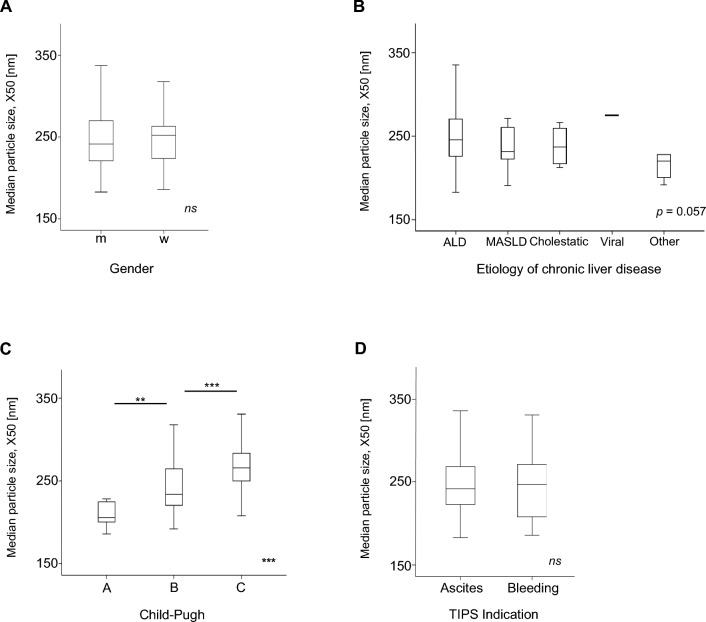
Enlarged particle size correlates with higher Child–Pugh class The median particle size X50 of the EVs of the study cohort correlated with gender (**A**) and the etiology of the chronic liver disease including ALD (alcohol-related liver disease), MASLD (metabolic dysfunction-associated steatotic liver disease), cholestatic, viral, and other causes (**B**). Increased median particle size were associated with higher Child–Pugh class (**C**). The indication of TIPS insertion correlated with median particle size (**D**). *** *p* < 0.001, ns = no significance. Kruskal–Wallis ANOVA test was used.

First, we analysed whether the median particle size of the EVs in serum correlated with important clinical parameters of the study cohort. We first considered the gender of the study participants and found no difference in the median particle size between men and women (Fig. 1A). We then looked at the etiology of chronic liver disease and found no association, while a non-significant trend towards increased particle size was observed in alcohol-associated liver disease (ALD) (*p* = 0.057) (Fig. 1B). We then analysed whether the median particle size differed between Child–Pugh classes. Patients in Child Pugh B had significantly larger EVs compared to Child Pugh A and significantly smaller compared to Child Pugh C (*p* < 0.001) (Fig. 1 C). Patients undergoing TIPS placement for refractory/recurrent ascites showed no difference compared to bleeding as an indication for TIPS placement (Fig. 1D).

### The baseline EVs concentration is not linked to the child–pugh class

**Fig. 2:**
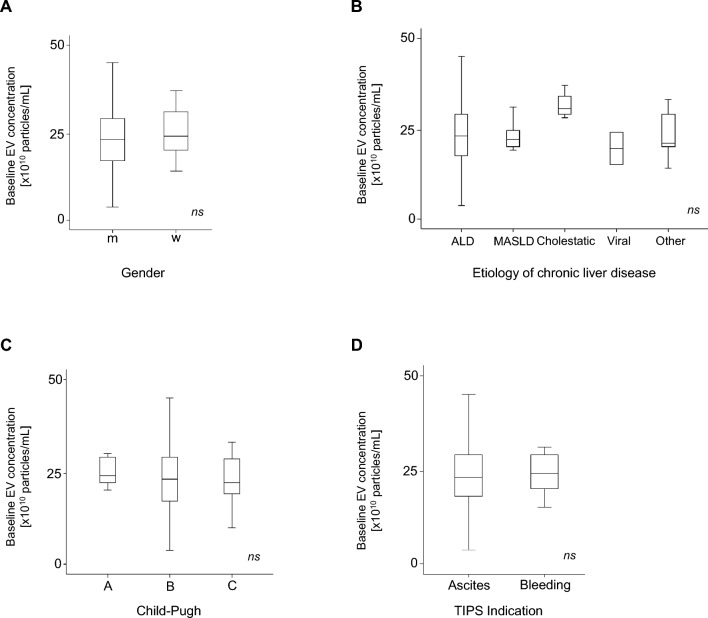
EVs concentration is not associated with advanced stage of chronic liver disease. Baseline EVs concentration was correlated to the gender (**A**), the etiology of the chronic liver disease including ALD (alcohol-related liver disease), MASLD (metabolic dysfunction-associated steatotic liver disease), cholestatic, viral, and other causes (**B**). Correlation of the baseline EVs concentration with the Child–Pugh class (**C**) and the TIPS indication showed no significance (**D**). ns = no significance. Kruskal–Wallis ANOVA test was used.

In the next step, we analysed the EVs concentrations in the serum of patients before TIPS insertion. Again, we found no difference between male and female patients (Fig. 2A). We then focused on the etiology of chronic liver disease and observed no difference between the different underlying pathologies (Fig. 2B). In contrast to the size of the EV, the concentration of the EVs showed no significant differences between the different Child–Pugh classes (Fig. 2C). The indication of the TIPS insertion also revealed no significance in the EVs concentration (Fig. 2D).

### The median particle size of circulating EVs correlates positively with the MELD and Child–Pugh-Score

**Fig. 3:**
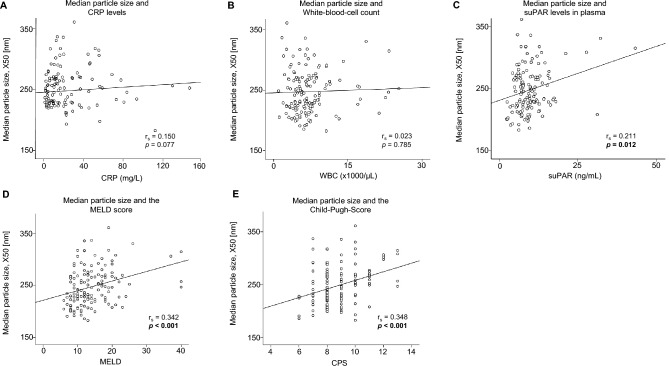
Correlation of the median particle size with inflammatory markers, MELD and Child–Pugh scores. The Median particle size was correlated with CRP levels (**A**), White-Blood-Cell (WBC) count (**B**), soluble urokinase plasminogen activator receptor (suPAR) (**C**), MELD (**D**) and the Child–Pugh score (**E**). Spearmann Rho correlation was used.

Next, we correlated the size of the EVs with key inflammatory parameters. We found no association with CRP levels (Fig. 3A) or white blood cell count (WBC) in the patients’ blood (Fig. 3B). In our previous publication, we demonstrated that soluble urokinase plasminogen activator receptor (suPAR) levels are an important predictor of survival in patients after TIPS placement. suPAR is a circulating marker of immune system activation and is associated with liver inflammation^14^. In correlation with the size of the EVs, we were able to detect a positive correlation (*p* = 0.012) (Fig. 3C). Subsequently, we analysed whether the size of the EVs also directly correlated with the MELD score and found a positive correlation (*p* < 0.001) (Fig. 3D). In addition, the size also correlated strongly positively with Child–Pugh score (*p* < 0.001) (Fig. 3E). As shown in Table 2, median particle size of EVs correlated with bilirubin (*p* < 0.001) and the INR (*p* < 0.001) in a positive fashion, while negative correlation with the platelet count (*p* = 0.015) was observed.

**Table 2:** Spearman correlation of EVs concentration and size with laboratory parameters.

	Baseline EVs concentration		Median particle size, X50	
	r_S_	*P* value	r_S_	*P* value
EVs cocentration (p/mL)		− 0.349	** < 0.001**	
EVs size, X50 (nm)	− 0.349	** < 0.001**		
suPAR	− 0.055	0.513	0.211	**0.012**
MELD	− 0.189	**0.025**	0.342	** < 0.001**
Sodium	0.113	0.300	− 0.062	0.569
Creatinine	− 0.136	0.108	0.035	0.682
Bilirubin	− 0.098	0.247	0.358	** < 0.001**
Albumin	− 0.077	0.483	− 0.068	0.534
INR	− 0.258	**0.002**	0.363	** < 0.001**
Platelets	0.320	** < 0.001**	− 0.205	**0.015**
ALT	− 0.063	0.456	− 0.055	0.518
WBC	0.232	**0.006**	0.023	0.785
CRP	− 0.058	0.493	0.150	0.077

EV, extracellular vesicles; suPAR, soluble urokinase plasminogen activator receptor level; MELD, model of end stage liver disease; INR, international normalized ratio; ALT, alanine transaminase; WBC, white blood cells; CRP, c-reactive protein.

Significant values are in bold.

### The baseline EVs concentration correlates with WBC in patients undergoing TIPS

**Fig. 4:**
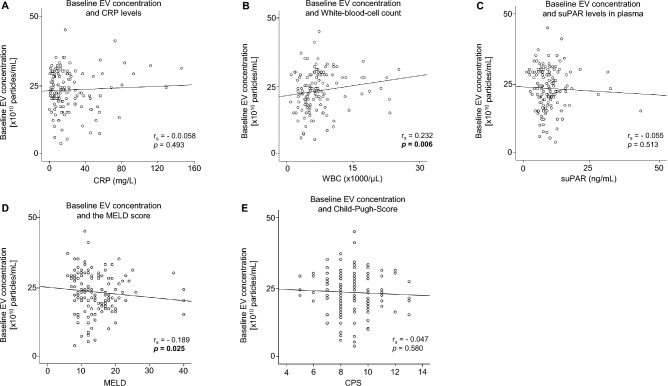
Correlation of the baseline EVs concentration with inflammatory markers, MELD and Child–Pugh scores. The baseline EVs concentration was correlated with CRP levels (**A**), White-Blood-Cell (WBC) count (**B**), soluble urokinase plasminogen activator receptor (suPAR) (**C**), MELD (**D**) and the Child–Pugh score (**E**). Spearmann Rho correlation was used.

Analysis of the baseline EVs concentration alone showed no correlation with the CRP levels in the serum (Fig. 4A). In contrast, the concentration of EVs correlated with the WBC count of patients undergoing TIPS (*p* = 0.006) (Fig. 4B). However, no association could be identified between suPAR levels and the concentration of EVs in the analysis (Fig. 4C). In the analysis of the MELD score, a positive correlation with the concentration of EVs was observed (*p* = 0.025) (Fig. 4D). However, no association was found in the analysis of the Child–Pugh-Score (Fig. 4E). Regarding the correlation with the important blood parameters, serum EVs concentration correlated positively with platelet (*p* < 0.001) and negatively with blood INR (Table 2).

### The size of the EVs is an independent predictive marker for transplantation-free survival in patients after TIPS placement

**Fig. 5:**
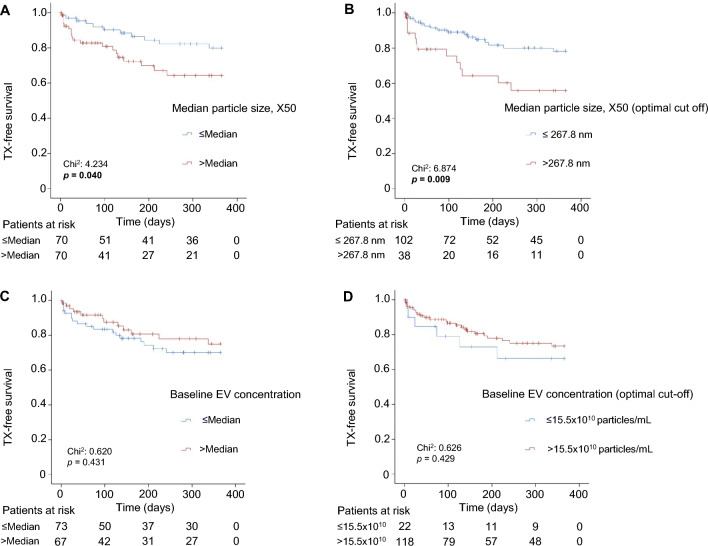
Enlarged particle size is associated with decreased 1-year transplant-free survival. Cumulative estimates of transplant-free survival of patients undergoing TIPS when median EVs size was dichotomized according to the median (242.5 nm) (**A**) or the optimized cut-off (267.8 nm) (**B**). Baseline EVs concentration was not predictive of post-TIPS transplantation (TX)-free survival when dichotomized according to the median (23 × 10^10^ particles/mL) (**C**) or the optimal cut-off value (15.5 × 10^10^ particles/mL) (**D**). The number of patients at risk is shown under the Caplan Maier curves. Log Rank test was used.

Next, we analyzed the impact of EVs characteristics on patient survival. The endpoints considered were death or liver transplantation (TX), so we focused on transplantation-free survival. Within follow-up of one year, 22 patients died, and 8 underwent transplantation. To assess whether the median particle size of the EVs in serum was associated with patient outcome, we first grouped the patients into low and high based on the median (242.5 nm) and found that patients with enlarged particle size had a significantly worse TX-free survival compared to patients with smaller EVs (*p* = 0.040) (Fig. 5A). We then calculated the optimal cut-off value of 267.8 nm (see Patients and Methods for details) and found an even stronger significant difference in TX-free survival (Fig. 5B). In univariate Cox regression, EVs greater than the optimal cut off value were strongly predictive of TX-free survival [HR: 2.539 (95% CI 1.232–5.23), *p* = 0.012, Table 3]. For comparison with the MELD score as a well-established prognostic marker, we also calculated the univariate Cox regression (HR: 1.070 [95% CI 1.026–1.116], *p* = 0.002, Table 3). We then performed multivariable Cox regression analysis and identified the median particle size of the EVs greater than the optimal cut-off value as an independent prognostic marker for TX-free survival in patients undergoing TIPS placement (HR: 2.204[95% CI 1.066–4.559], *p* = 0.033, Table 3). The MELD score remained an independent predictor in multivariable Cox regression (adjusted HR: 1.066 [95% CI 1.019–1.115], *p* = 0.006, Table 3). Subsequently, we analysed whether the concentration of serum EVs correlated with TX-free survival of the patients. We therefore grouped the patients according to the median of 23 × 10^10^ particles/mL and found no difference between patients with increased and decreased concentrations (Fig. 5C). The calculation of the optimal cut-off value of 15.5 × 10^10^ particles/mL (see Patients and Methods for details) also showed no significant difference, so that it can be concluded that the baseline concentration of EVs does not correlate with survival of patients after TIPS placement (Fig. 5D).

**Table 3:** Uni- and multivariable Cox-regression analyses for transplant-free survival.

	Univariate regression models		Multivariable regression model	
Parameter	*P* value	Hazard ratio (95% CI)	*P* value	Hazard ratio (95% CI)
Baseline EVs concentration (dichotomized according to the optimal cut-off value)	0.432	0.698 (0.285–1.709		
Median particle size, X50 (dichotomized according to the optimal cut-off value)	**0.012**	2.539 (1.232–5.233)	**0.033**	2.204 (1.066–4.559)
MELD Score (continuous variable)	0.002	1.070 (1.026–1.116)	**0.006**	1.066 (1.019–1.115)

The optimal cut-off for the baseline EVs concentration: 15.5 × 1010 particles/mL.

The optimal cut-off for the median particle size, X50: 267.8nm.

Significant values are in bold.

## Discussion

In this study, we performed a detailed analysis of the concentrations and characteristics of EVs in a cohort of 141 patients undergoing TIPS placement. We obtained peripheral blood prior intervention. Our results showed that patients with a higher Child–Pugh class had significantly larger particle size of the EVs in the serum, and patients with enlarged EVs had significantly worse survival. Despite a moderate correlation of EVs size with established post-TIPS survival parameters (MELD and Child–Pugh scores), enlarged EVs particle size remained an independent predictor of transplant-free survival after TIPS placement, even after adjustment for MELD in a multivariable Cox regression analysis.

Despite technical advanced over the past 30 years that have reduced immediate procedural complications, careful patient selection remains essential to balance urgency and effectiveness against potential contraindications^15^. Studies have revealed that systemic inflammation is critical to for the prognosis of patients with PH. In this process, viable bacteria and bacterial byproducts translocate through the gut mucosa into systemic circulation, leading to secondary systemic inflammation^21,22^. This leads to an elevated secretion of cytokines and reactive proteins, which increase the permeability of the blood–brain barrier and thus promote hepatic encephalopathy and acute decompensation of liver cirrhosis^23^. It has been demonstrated that patients with an elevated serum level of CXCL9, soluble TNF-alpha-receptor I, CXCL11, or soluble urokinase plasminogen activator receptor (suPAR) have a reduced survival after TIPS placement^14,24–26^. In our cohort, EVs size correlated with the immune activation marker suPAR but not with clinical routine parameters of inflammation and acute phase, such as WBC and CRP^27^.

EVs are lipid bound vesicles secreted by various cell types, such as endothelial cells, immune cells, hepatocytes, and hepatic stellate cells, into the extracellular space under both normal and pathological conditions^28^. Their potential for early diagnosis and prognosis has become a major research focus in liquid biopsy, as they are present in nearly all biological fluids (e.g., blood, ascitic fluid, saliva, urine)^29,30^. The content loaded from EVs varies depending on their cellular source. EVs derived from T cells and monocytes are rich in pro-inflammatory cytokines, including TNF-alpha, IL-2, and IL-6^31,32^. It is tempting to speculate that determining EVs size may harbors information on the inflammatory profile in advanced liver disease that is not captured by routine clinical parameters.

Most of the patients in our study cohort suffered from alcohol-related liver disease (ALD). Alcohol exposure increases the number of circulating liver-derived EVs, which in turn promotes inflammation and fibrosis^33^. Studies demonstrated that elevated levels of microRNAs miR-122 and miR-155 are found in plasma of ALD patients and were associated with EVs^34^. These hepatocyte-derived EVs increase the secretion of pro-inflammatory cytokines in monocytes^35^. Another study identified CD40 ligand (CD40-L) in EVs after alcohol exposure, which plays an important role in macrophage activation^36^. In our results, the size of the EVs was prognostically significant, but not the concentration. This may indicate that different structures are loaded in the EVs depending on its size, and thus have different effects on immune response and prognosis of the patient.

In addition to enhancing inflammation, EVs have also been shown to promote liver fibrosis in the context of steatotic liver disease including NAFLD and ALD^37^. Hepatic stellate cells (HSC), which are responsible for the initiation and maintenance of fibrosis, have been shown to secrete EVs upon activation. EVs contain a variety of profibrotic proteins and nucleic acids^38–40^. Liver injury further increases the secretion of these EVs^41^. On the other hand, EVs from mesenchymal stem cells (MSC) have been shown to exert a protective effect on the liver. In the mouse model with liver injury, the application of MSC-derived EVs led to an activation of regenerative mechanisms in the liver, a stimulation of hepatocytes, a reduction of apoptosis and hepatic inflammation^42–44^. As we did not investigate the content and source of EVs in this study, we can only speculate about cellular source and biological consequences of larger circulating EVs in our setting.

Our study demonstrated that although the overall concentration of EVs did not have prognostic relevance, our data interestingly showed that EV concentration correlated positively with platelet count and negatively with the INR of the patients. One possible explanation for this finding is that platelets are a major source of EVs^45^. Therefore, platelet count may be directly associated with EV concentration. Patients with advanced liver disease often exhibit elevated INR levels due to impaired hepatic synthetic function. Additionally, portal hypertension commonly leads to hypersplenism, resulting in thrombocytopenia^46^. This could explain why patients with elevated INR levels also show reduced EV concentrations. Further studies are needed to clarify this issue conclusively.

Further limitations must be taken into account when considering the results of the present study. Due to the relatively small number of patients, the statistical power is limited. Therefore, detailed subgroup analyses could not be performed. It is particularly important to highlight that the vast majority of patients in our study cohort suffered from ALD. Due to the low number of patients with other etiologies, such as viral hepatitis, meaningful subgroup analyses could not be performed. Therefore, it remains unclear whether the reported findings are applicable to all causes of liver disease or are specific to alcohol-related liver disease. Further studies are needed to address this question.

Due to the retrospective study design, it was not possible to achieve complete standardization in the detailed assessment of clinical factors, such as the progression of comorbidities such as heart failure. In addition, patient selection for TIPS insertion in clinical practice were considering Child–Pugh and MELD scores, presumably introducing selection bias.

In conclusion, we have shown that larger circulating EVs size is associated with a more advanced stage of chronic liver disease and is an independent predictive marker for TX-free survival after TIPS placement. Larger scaled prospective studies are needed to confirm our findings and to identify patients at particularly high risk after TIPS placement in detailed subgroup analyses.

## Data Availability

For all data requests, please contact the corresponding author Christoph Roderburg.
